# Real-Time Quaking-Induced Conversion Assay Applied to the Italian Chronic Wasting Disease Monitoring Plan: Comparison of Classical and Innovative Diagnostic Methods

**DOI:** 10.3390/pathogens14101053

**Published:** 2025-10-18

**Authors:** Maria Mazza, Alessandra Favole, Valentina Campia, Barbara Iulini, Romolo Nonno, Ciriaco Ligios, Davide Pintus, Simone Peletto, Cristina Casalone, Cristiano Corona, Elena Bozzetta, Pier Luigi Acutis

**Affiliations:** 1 European Reference Laboratory for TSEs—Italian Reference Laboratory for TSEs, Istituto Zooprofilattico Sperimentale del Piemonte, Liguria e Valle d’Aosta, Via Bologna 148, 10154 Turin, Italy; alessandra.favole@izsplv.it (A.F.); valentina.campia@izsplv.it (V.C.); barbara.iulini@izsplv.it (B.I.); simone.peletto@izsplv.it (S.P.); cristina.casalone@izsplv.it (C.C.); cristiano.corona@izsplv.it (C.C.); elena.bozzetta@izsplv.it (E.B.); pierluigi.acutis@izsplv.it (P.L.A.); 2 Istituto Superiore di Sanità, Viale Regina Elena 299, 00161 Rome, Italy; romolo.nonno@iss.it; 3 Istituto Zooprofilattico Sperimentale della Sardegna, Via Duca degli Abruzzi 8, 07100 Sassari, Italy; ciriaco.ligios@izs-sardegna.it (C.L.); davide.pintus@izs-sardegna.it (D.P.)

**Keywords:** CWD, RT-QuIC, TSE, prion diseases, TSEs diagnosis, deer

## Abstract

CWD surveillance and diagnosis are important issues in Europe since its detection in Norway, as some of its strains, like that of classical scrapie, are contagious. In addition, there are concerns as several matters about CWD are not yet known. Although diagnostic methods for the active surveillance in bovine and small ruminants have been able to detect the European CWD strains, a retrospective study on Italian wild red deer (*Cervus elaphus*) samples was performed to compare the results obtained from rapid screening tests, authorized according to EU Regulation 999/2001, and the RT-QuIC, a highly sensitive method in the detection of prion disease infection. A total of one hundred brainstems and medial retropharyngeal lymph nodes were selected out of those received from the CWD Italian surveillance system. Confirmed CWD-positive and -negative samples were included in the study as controls. All of the samples were first tested with the HerdChek BSE–Scrapie Antigen Test and then using the RT-QuIC. The rapid test was negative in all brainstem and lymph node samples. RT-QuIC analyses showed only one red deer brainstem sample positive for seeding activity, while all lymph nodes were negative, including the one from this case. This positive brainstem sample was then re-extracted and retested using two different recombinant prion protein substrates (Ha90-231; BV23-231) and their different batches from the first analyses. Seeding activity was consistently confirmed across both substrates and extractions, with positive signals detected down to dilutions of 10^−4^ using rPrP Ha90-231 and as low as 10^−6^ with rPrP BV23-231. The additional diagnostic investigations performed on this red deer using the alternative rapid test (TeSeE SAP Combi), Western blot, and immunohistochemistry showed negative results both in the brainstem and lymph nodes. This study showed that overall, the results obtained with the HerdChek BSE–Scrapie Antigen Test and RT-QuIC agree except in one case. Our findings highlight the potential of the RT-QuIC method to detect very low levels of PrP^Sc^-associated seeding activity that may escape detection using classical methods. While seeding activity does not always equate to infectivity, only a bioassay will confirm the real disease status of this Italian case. These findings support the integration of RT-QuIC as a powerful complementary tool within existing surveillance frameworks to strengthen early detection and diagnostic accuracy.

## 1. Introduction

Chronic Wasting Disease (CWD) is a fatal neurodegenerative disease that affects different cervid species (elk, deer, moose, and reindeer), and its most predominant clinical sign is emaciation. It is part of Transmissible Spongiform Encephalopathies (TSEs) or prion diseases that affect animals and humans, including bovine spongiform encephalopathy (BSE) in cattle, Scrapie in sheep and goats, and Creutzfeldt–Jakob disease (CJD) in humans.

CWD, along with other prion diseases, is due to the misfolded pathogenic form (PrP^Sc^) of the host-encoded cellular prion protein (PrP^C^). Prion accumulation in the central nervous system (CNS) leads to neurodegeneration and, eventually, death [1,2,3,4].

CWD, like classical Scrapie, is a highly contagious disease under natural conditions; horizontal transmission occurs between cervids through direct contact and environmental exposure. The disease affects not only the CNS but also the lymphoreticular system. PrP^Sc^ has been found in the saliva, feces, and urine of CWD-infected animals, indicating a possible environmental spread of the infectious agent. These findings suggest a high risk of exposure for other animal species, especially since prions can survive for many years in soil [5,6,7,8,9]. Although no transmission of American CWD strains has been shown in transgenic mice expressing human PrP, a potential risk of transmission to humans cannot yet be ruled out as many aspects of the disease need to be further investigated.

CWD was first identified in 1967 in a mule deer (*Odocoileus hemionius*) in Colorado and since then, it has been detected in 30 states in the United States and in four provinces in Canada (Saskatchewan 1996, Alberta 2005, Quebec 2018, and Manitoba 2021), in South Korea, and more recently in Norway, Finland, and Sweden [10,11,12].

The number of cervids currently infected with CWD is very high in the U.S., with approximately 30 million white-tailed deer involved. From a public health point of view, there are more than 11 million deer hunters, and it has been estimated that up to 15,000 CWD-infected cervids are consumed annually. Moreover, the geographic areas with high prevalence of CWD cases overlap with other species that may be susceptible to CWD via contact with cervids or CWD-contaminated environments [13].

CWD is present in Europe, although with a much lower prevalence than that reported in the U.S. The first case of CWD was identified in 2016 in a free-ranging reindeer (*Rangifer tarandus*) in Norway [11]. To prevent the spread of CWD within the EU and/or to control the disease where it occurs, the European Commission implemented a CWD surveillance program in cervids by using diagnostic rapid tests approved by the Commission Regulation (EU) 2017/1972 and supported by data obtained from US CWD cases [14]. As a result of this monitoring, a total of 44 cases of CWD have been detected to date, 37 of which were reported in Norway and affected three cervid species: 21 reindeer (*Rangifer tarandus*), 13 moose (*Alces alces*), and three red deer (*Cervus elaphus*), and three cases in Finland and four in Sweden, all in moose [15,16]. The origin of CWD in Europe is still unknown. Molecular and biological assays showed that prion strains present in European CWD cases were multiple and different from those circulating in North America, resulting in different PrP^Sc^ distribution patterns in host tissues [16,17,18,19]. A PMCA study performed recently on Norwegian CWD cases showed (i) the presence of PrP^Sc^ in different peripheral tissues, including muscles, and (ii) infectivity of the lymph node and muscle of a moose when experimentally transmitted to bank voles [20]. These recent findings agree with the results shown in previous transmission studies performed on CWD strains from North America. Following this evidence, many perplexities have arisen in the European scientific community about the ability and, notably, the sensitivity of diagnostic methods to detect European CWD strains.

The diagnostic data obtained from the survey conducted in Europe, as well as findings coming from comparative studies, confirmed the validity of the diagnostic system applied across the Member States to detect European CWD strains [21]. It is now well established that the best marker for the diagnosis of CWD and all other TSEs is the pathological isoform of the prion protein (PrP^Sc^); therefore, their diagnosis can only be assessed post-mortem at the level of the CNS, where the etiological agent accumulates in relatively high concentrations. PrP^Sc^ is also present in quantities too low to be detected using conventional diagnostic methods in more easily accessible biological samples. To overcome this limitation, two useful methods have been developed and successfully applied by researchers studying prions: protein misfolding cyclic amplification (PMCA) and Real-Time Quaking-Induced Conversion (RT-QuIC) [22,23].

Although diagnostic methods applied for active surveillance in cattle and small ruminants have been able to detect European strains of CWD, to be able to perform an increasingly accurate diagnosis of this disease, we carried out a retrospective study on Italian wild deer samples from the voluntary surveillance established by the Italian Ministry of Health, comparing the results obtained using the authorized rapid screening test and the Real-Time Quaking-Induced Conversion (RT-QuIC) method, well known to be an innovative approach for its very high sensitivity on both prion and prion-like diseases [24,25]. Even if PMCA and RT-QuIC are characterized by the same sensitivity, the use of the latter method in our study was based on many reasons: lower costs, faster analysis, simpler performance and, above all, for biosafety reasons, as, unlike PMCA, it does not generate infectious amplification. RT-QuIC can rapidly detect sub-infectious doses of PrP^Sc^ as prion seeding activity and has been successfully used to detect multiple human, cervid, ovine, hamster, and mouse prion strains in a variety of biological tissues with a similar sensitivity, if not even greater, than that of biological tests. To date, a variety of clinical samples, including cerebrospinal fluid (CSF), urine, feces, saliva, blood, and *post-mortem* and *ante-mortem* tissues, have been evaluated for the diagnosis of CWD using the RT-QuIC [26,27,28,29].

## 2. Materials and Methods

### 2.1. Animals and Samples

One hundred wild adult deer (*Cervus elaphus*), found dead, were selected from those received in the frame of the Italian CWD monitoring plan. The brainstem and medial retropharyngeal lymph node (RPLN) samples were collected from these cases by the local veterinary services and sent to the laboratories in charge of the Italian TSEs surveillance. These samples were then longitudinally cut into two halves: one was frozen at 80 °C, and the other was fixed in 10% buffered formaldehyde solution for confirmatory immunohistochemistry (IHC) and shipment to our center for diagnostic screening by rapid test. The frozen samples from each animal were submitted to the rapid test HerdChek BSE-Scrapie Antigen Test Kit, EIA (One IDEXX Drive, Westbrook, ME, USA) and subsequently to the RT-QuIC assay.

To evaluate the performance of the RT-QuIC method to detect the CWD strains circulating in Europe, 13 brains from CWD-positive cases were included in the study: six Norwegian samples (five moose and one deer); four Finnish moose; and three Swedish moose. Two brain samples from Canadian moose, which tested positive and negative for CWD, were used as controls. To avoid cross-contamination with tissues and homogenates from positive controls, resulting in false positive results, careful attention was taken during all stages of sample processing by the laboratory, using disposable materials and monitoring the specificity of amplification reactions by including negative control samples in all analysis sessions.

### 2.2. Study Protocol

Based on the protocol established in this study, all samples that tested positive or doubtful in the applied diagnostic tests were retested with both the Idexx test, RT-QuIC, and, in addition, the rapid test TeSeE™ SAP Combi Kit (Bio-Rad, Hercules, CA, USA) and two confirmatory tests, Western Blot (WB) and Immunohistochemistry (IHC). A genetic analysis of the *PRNP* gene was also performed on those samples that possibly tested positive. This workflow was defined according to Regulation CE 999/2001.

### 2.3. TSE Diagnostic Investigations

#### Rapid Tests

The primary screening test applied to analyze the deer samples selected for this comparative study was the HerdChek BSE-Scrapie Antigen Test Kit, EIA (Idexx), and for a secondary analysis of the positive samples, the TeSeE^TM^ SAP Combi Kit (Bio-Rad), based on different principles. The latter test is based on a homogenate digestion step of PrP^C^ with proteinase K (PK) to select PrP^Sc^, which is partially resistant to PK action due to its β-sheet structure and its aggregation formation; the HerdChek BSE-Scrapie Ag test does not involve any digestion with PK, but it uses a particular ligand that can capture PrP^Sc^ by a specific conformational recognition of PrP^Sc^ aggregates.

### 2.4. HerdChek BSE-Scrapie Antigen Test (Idexx)

The test was carried out according to the manufacturer’s instructions. Briefly, 120 μL of homogenate was mixed with 30 μL of the working plate diluent solution (D1 and D2), and 100 μL of the mixture was loaded onto the antigen-capture plate and shaken for 45 min at room temperature. After washing, the plate was incubated in 100 μL of conditioning buffer (CB) for 10 min. Abnormal PrP was detected using 100 μL of the kit conjugated anti-PrP antibody, conjugate concentrate (CC) (incubation of 45 min), and then visualized with 100 μL TMB (tetramethylbenzidine) for 15 min of incubation in the dark and absorbance read at 450 nm and 620 nm. The interpretation of sample results is based on the absorbance of the sample. Samples with OD values less than the cut-off value are considered negative; samples with OD values greater than or equal to the cut-off are classified as positive. Calculation of the cut-off value was carried out according to the manufacturer’s instructions.

### 2.5. TeSeE^TM^ SAP Combi Kit (Bio-Rad)

This rapid test is an ELISA sandwich technique. The analyses were performed as reported previously [21]. Briefly, 250 μL of the homogenate sample was incubated for 10 min at 37 °C with 250 μL of denaturing solution (buffer A), containing PK. Digestion was stopped by the addition of 250 μL of clarifying solution (buffer B). PrP^Sc^ was recovered as a pellet after the micro test-tubes were centrifuged at 20,000× *g* for 5 min at room temperature. The supernatant was discarded, and the tubes were dried. Finally, the pellet was denatured in 25 μL resolving buffer (5 min at 100 °C), then diluted with 125 μL sample diluent reagent R6, before 100 μL of it was distributed into the ELISA wells. The immunodetection part was performed for 30 min at 37 °C, washes, 100 μL of conjugate solution R7, and incubation for 30 min at between 2 and 8 °C, washes, before 100 μL of the enzymatic revelation solution (R8 + R9) was applied for 30 min in darkness at room temperature. The revelation process was stopped by adding 100 μL of stop solution (R10) to each well, and the absorbance was read at 450 nm and 620 nm. Samples with an OD lower than the cut-off value are considered to be negative; samples with an OD greater than or equal to the cut-off value are considered to be positive. Calculation of the cut-off value was carried out according to the manufacturer’s instructions.

### 2.6. Real-Time Quaking-Induced Conversion (RT-QuIC) Assay

RT-QuIC is a seeded conversion assay that amplifies minute amounts of misfolded prion protein (PrP^Sc^) by converting bacterially expressed recombinant PrP (rPrP^Sen^) into amyloid fibrils, monitored in real time by Thioflavin-T (ThT) fluorescence measurements [23,24].

Brainstem and medial retropharyngeal lymph node homogenates (BHs and RPLNHs) (10%, wt/vol) were prepared as previously described [30] and stored at −80 °C. For RT-QuIC analysis, BHs and RPLNHs were serially diluted in 0.1% SDS (Sigma-Aldrich, Merck KGaA, Darmstadt, Germany)–N2 (Gibco, Thermo Fisher Scientific, Waltham, MA, USA)–PBS as previously reported [23,24]. The RT-QuIC reaction mix was composed of 10 mM phosphate buffer (pH 7.4), 300 mM NaCl, 10 mM ThT, 1 mM EDTA, an1 mg/mL of rPrP^Sen^. Aliquots of 98 μL reaction mix were loaded into 96-well plates with clear bottoms (Nalgene Nunc International) and seeded with 2 μL of BH and RPLNLH dilutions (from 10^−3^ to 10^−8^). Normal and affected deer BH dilutions were used as negative and positive controls, respectively. The plate was then sealed with a plate-sealer film (Thermo Fisher Scientific, Rochester, NY, USA) and incubated for 90 h at 42 °C in a BMG Labtech (BMG Labtech, Ortenberg, Germany) FLUOstar Omega plate reader with cycles of 1 min of shaking (700 rpm double orbital) and 1 min of rest throughout incubation. ThT fluorescence measurements (excitation, 450 ± 10 nm; emission, 480 ± 10 nm; bottom read) were recorded every 45 min.

#### 2.6.1. RT-QuIC Data Analysis

RT-QuIC reactions were deemed acceptable when the negative controls remained below the threshold and the positive CWD (*American moose*) controls amplified within expected time frames (~12 h). A 70 h time point was chosen based on multiple (n = 5) repeat runs in which no spontaneous conversions of the substrate in negative control seeded reactions were observed. RT-QuIC fluorescence readings were analyzed as previously described [23,24], and data are displayed as the average of four technical replicates. Briefly, a well was considered positive when the signal crossed the threshold (mean of negative control wells + standard deviation), and positivity required ≥2/4 technical replicates to exceed the threshold within the runtime. RT-QuIC amyloid formation rates were calculated as the inverse of the lag time before fluorescence reached the threshold.

#### 2.6.2. Recombinant Prion Protein Purification

Syrian golden hamster [amino acids 90–231; GenBank accession no. K02234] and bank vole [amino acids 23–231; GenBank accession number AF367624] prion protein genes were ligated into the pET41 vector (MilliporeSigma, Merck KGaA, Darmstadt, Germany). *Escherichia coli* carrying this vector was grown in Luria broth (LB) medium in the presence of kanamycin and chloramphenicol. rPrP^Sen^ expression was induced using the Overnight Express Autoinduction system 1 (Novagen, MilliporeSigma, Merck KGaA, Darmstadt, Germany) and BugBuster master mix (Novagen, MilliporeSigma, Merck KGaA, Darmstadt, Germany) to isolate inclusion bodies. Following solubilization of the inclusion bodies in 8 M guanidinium-HCl, the denatured protein was purified under 6 M guanidinium-HCl denaturing conditions using nickel nitrilotriacetic acid (Ni-NTA) super flow resin (Qiagen, Hilden, Germany) with an AKTA fast protein liquid chromatography instrument (GE Healthcare, Chicago, IL, USA). The rPrP^Sen^ was subjected to on-column refolding using a linear gradient into phosphate buffer and then eluted using an imidazole gradient as previously described [23,24]. The purified protein was extensively dialyzed into 10 mM sodium phosphate buffer (pH 5.8). Then, following filtration (0.22 mm syringe filter; Thermo Fisher Scientific, Paisley, UK), a concentration measurement by absorbance at 280 nm was performed, and the rPrP^Sen^ was stored at −80 °C.

### 2.7. Lipids Ethanol Extraction from Brain Homogenates

Lipid extraction was performed as reported by Hoover et al. [31], using a slightly modified protocol. Briefly, 5 µL of 10% brain homogenates from CWD controls (positive and negative) and deer 9 were incubated in 45 µL of 96% ethanol for 5 min at room temperature. The samples were pelleted via centrifugation at 21,000× *g* for 7 min, and the supernatant was removed. The pellets were then dissolved in 5 µL of 0.1 SDS/PBS buffer and analyzed using the RT-QuIC assay.

### 2.8. Confirmatory WB

Ten percent (*w*/*v*) homogenates of brainstem tissue and medial retropharyngeal lymph nodes were prepared in lysis buffer [10% N-lauroylsarcosine diluted in Tris-buffered saline (TBS), pH 7.4] and clarified via centrifugation at 22,000× *g* for 20 min at 10 °C. Next, 1 mL of each supernatant was digested by proteinase k (40 µg per ml) at 37 °C for 1 h. The samples were then centrifuged at 215,000× *g* for 1 h at 10 °C; the pellets were dissolved in 50 μL of Laemmli buffer and 50 μL of distilled water. A denaturation step of the protein extract was performed at 99 °C for 5 min. Thereafter, 10 μL (corresponding to 10 mg of tissue) of this extract was subjected to sodium dodecyl sulfate–polyacrylamide gel electrophoresis with Nu-PAGE Bis-Tris mini-gels (Invitrogen, Thermo Fisher Scientific, Waltham, MA, USA) and then transferred onto polyvinylidene difluoride membranes using a Trans-Blot Turbo Transfer System (Bio-Rad). PVDF membranes were blocked for 1 h at room temperature in a blocking buffer [5% Bovine Serum Albumin diluted in TBS, pH 7.4]; the blots were then incubated for 1 h at room temperature with the monoclonal antibody (mAb) anti-PrP Sha31 (aa148-155) from SpiBio, France, diluted 1:2000 in TBS-Tween20 (TBS-T). Immunosignals were revealed with an alkaline phosphatase-conjugated goat anti-mouse immunoglobulin G (0.1 µg per mL) (Invitrogen, Thermo Fisher Scientific, Waltham, MA, USA), and immuno-reactivity was visualized using a chemiluminescent reaction with Novex^®^ AP Chemiluminescent Substrate CDP-Star^®^ (Invitrogen, ThermoFisher Scientific, Waltham, MA, USA). The images of the blots were captured with a gel documentation imaging system iBright (ThermoFisher Scientific Imaging System, Waltham, MA, USA). Samples were classified as positive when at least the di-glycosylated band of PrP^Sc^ was present.

### 2.9. Confirmatory IHC

Formalin-fixed, paraffin-embedded tissue sections from brainstem and retropharyngeal lymph nodes were dewaxed in a xylene substitute and rehydrated through graded alcohols. The sections were then treated with 98–100% formic acid for 25 min to enhance epitope exposure, followed by antigen retrieval in citrate buffer (pH 6.1) using an autoclave cycle (30 min at 121 °C). After cooling to room temperature, the slides were washed in tap water, and endogenous peroxidase activity was blocked with 3% hydrogen peroxide in methanol for 20 min. The slides were then washed again in tap water and stored overnight in distilled water at 5 ± 3 °C. The following day, sections were equilibrated in TBST (pH 7.4) and incubated with normal horse serum (VECTASTAIN^®^ ABC-HRP Kit, PK-4002, Vector Laboratories, Burlingame, CA, USA) for 20 min to block non-specific binding. Six monoclonal anti-PrP primary antibodies were diluted in TBST (F99/97.6.1 at 1:1000; F89/160.1.5 at 1:1000; P4 at 1:500; L42 at 1:250; 6C2 at 1:500; 2G11 at 1:500) and incubated for 1 h at room temperature. After washing, a biotinylated anti-mouse secondary antibody (1:200 in TBST) and the avidin–biotin complex (VECTASTAIN^®^ ABC-HRP Kit, PK-4002, Vector Laboratories, Burlingame, CA, USA) were applied sequentially, each for 30 min. Detection was carried out using DAB chromogen (Agilent DAKO, K3468, Carpinteria, CA, USA), with development monitored microscopically to optimize incubation time and minimize background staining. The reaction was stopped in distilled water. Slides were counterstained with hematoxylin, dehydrated through graded ethanol and a xylene substitute, and mounted with a permanent mounting medium.

### 2.10. Genetic Analysis

Genomic DNA was isolated using the Qiagen Tissue DNA extraction kit (Qiagen, Hilden, Germany). The DNA segments corresponding to the complete open reading frame (ORF) of the PRNP gene (771 bp) were PCR-amplified using primers p78 (+) (5′ TAA GTG GGC ATA TGA TGC TG 3′), p79 (-) (5′ GGG CTG CAG GTA GAC ACT C 3′), p61 (+) (5′ AAC CAA CAT GAA GCA TGT GG 3′), and p60 (-) (5′ GAT AGT AAC GGT CCT CAT AG 3′). PCR was performed according to a previously described protocol [32]. The DNA sequencing reactions were carried out using a BigDye Terminator Cycle Sequencing Kit (Thermo Fisher Scientific, Waltham, MA, USA), and the DNA sequences were analyzed on a SeqStudio Genetic Analyzer (Thermo Fisher Scientific, Waltham, MA, USA). Sequence electropherograms were visualized using Sequencing Analysis software v. 5.2 and manually inspected for quality. Finally, forward and reverse sequences were aligned using the SeqMan Ultra software v. 17.6.2 included in the Lasergene package (DNASTAR Inc., Madison, WI, USA) for polymorphism detection, as reported by Peletto et al. [33].

## 3. Results

The screening analyses carried out on the brainstem and medial retropharyngeal lymph nodes of Italian deer with the rapid test HerdChek BSE-Scrapie Antigen Test showed negative results for all cases included in this study.

**Figure 1:**
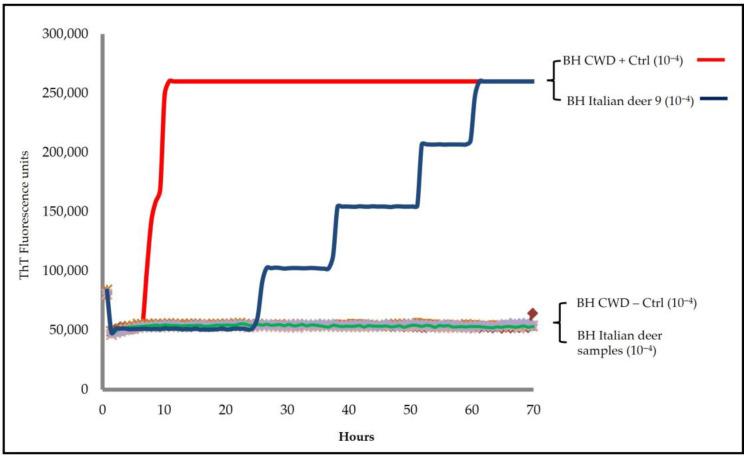
Representative RT-QuIC analysis of brainstem homogenates from Italian deer. RT-QuIC reactions for all Italian deer and positive and negative CWD controls were seeded with 2 μL of 10^−4^ BHs dilution and Ha90-231 as substrate.

The RT-QuIC protocol applied by our laboratory on European CWD samples and the Canadian CWD positive control showed the ability of this method to correctly identify all positive and negative CWD cases, thus revealing the high sensitivity and specificity performance of this amplification method (Supplementary Material Figure S1). The hundred Italian deer cases were then analyzed with the RT-QuIC method using two different recombinant prion protein substrates (rPrP^Sen^), hamster truncated (Ha90-231) and bank vole (BV23-231). Seeding activity was detected only in one animal (deer 9), exclusively in the brainstem, with a lag phase of approximately 20 h (Figure 1); all other brainstem and medial retropharyngeal lymph node samples were negative.

**Figure 2:**
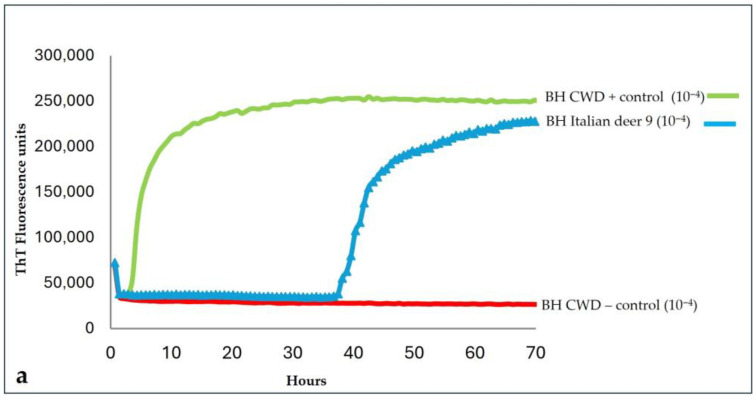

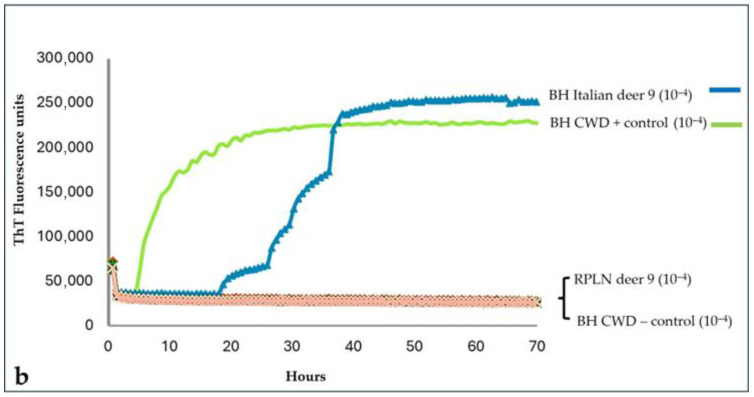
RT-QuIC analyses of (**a**) brainstem and (**b**) medial retropharyngeal lymph node (RPLN) homogenates from Italian deer 9. These homogenates, positive and negative CWD controls, were seeded with 2 μL of 10^−4^ BHs dilution and BV23-231 as substrate.

To exclude possible cross-contamination with positive controls, the brainstem and medial retropharyngeal lymph node from deer 9 were retested, starting a new sampling. The new RT-QuIC investigations with the BV23-231 substrate still showed seeding activity only in the brainstem, thus confirming the result of the first analysis (Figure 2a,b). Furthermore, a quicker amplification, in both fluorescence values and time, was observed in the brainstem homogenate of deer 9 using the BV23-231 substrate rather than the Ha90-231 substrate. Furthermore, the brain homogenate from this case was prepared using an ethanol-based extraction protocol to reduce the inhibitory effects of lipids, as previously described by Hoover et al. [31]. Ethanol-mediated lipid removal substantially increased the amplification efficiency of the homogenate from deer 9 compared with the untreated control (Supplementary Material, Figure S5).

**Figure 3:**
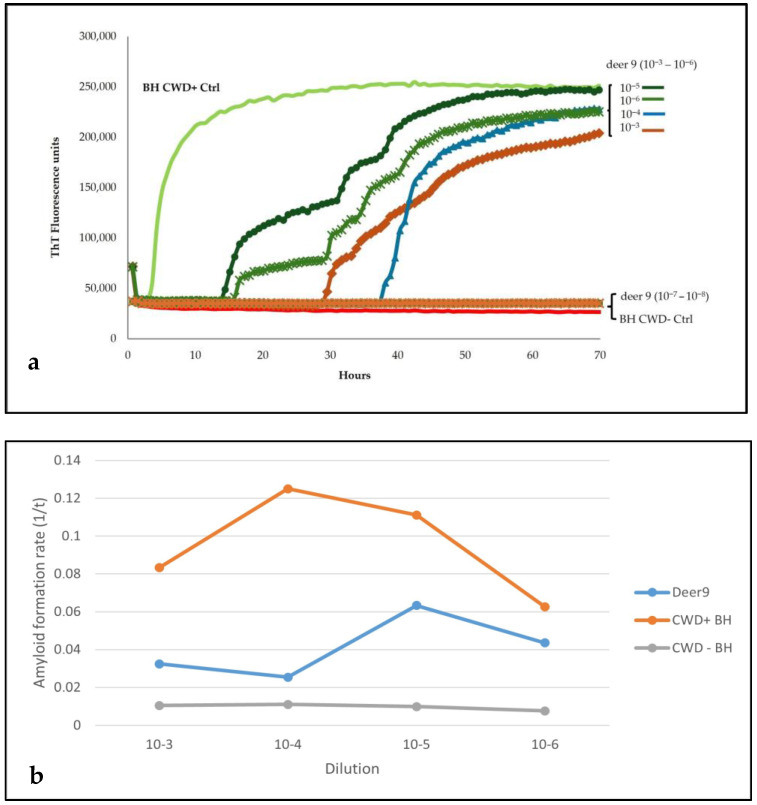
Sensitivity analysis of the brainstem homogenate from Italian deer 9. (**a**) The reactions were performed using BV23-231 and six different dilutions (10^−3^ to 10^−8^). Dilution 10^−4^ was applied for positive and negative CWD controls. (**b**) RT-QuIC amyloid formation rates of deer 9, CWD positive and negative controls, were shown as the inverse of the lag time versus sample dilutions.

Sensitivity analysis of the brainstem homogenate from this animal showed seeding activity until the dilution 10^−6^, while no amplification was present in dilutions 10^−7^ and 10^−8^ (Figure 3a,b).

The brainstem and RPLN homogenates from this case were retested with the HerdChek BSE-Scrapie Antigen Test and then subjected to the following diagnostic and genetic analyses:

1. TeSeE^TM^ SAP Combi Kit, as an alternative rapid test.
2. Confirmatory TSE analyses through WB and IHC.
3. *PRNP* gene sequencing.

Further diagnostic investigations using additional screening tests and confirmatory analyses performed through Western Blot and immunohistochemistry on this case did not show any signal of PrP^Sc^, neither in the brainstem or RPLN samples. Table 1 and Supplementary Figures S2–S4 summarize the results of the analyses performed on this case.

The alleles and genotypes of the *PRNP* gene at the seven codons investigated are reported in Table 2.

Unfortunately, limited information has been recovered on this animal: an adult male wild deer found dead in a region of northern Italy. The local veterinary services provided no information on the real reason for its death other than to rule out death by roadkill.

## 4. Discussion

The control and eradication of TSEs is a major challenge for the scientific community, as there are no methods available for an early diagnosis. In addition to the absence of an *ante-mortem* test, for some of them, such as Classical Scrapie and CWD, the scenario is even more critical, as they are contagious and can contaminate the environment through excreta, resulting in horizontal transmission to other species as well.

CWD status of an animal is currently evaluated *post-mortem* in the central nervous system, brain, and/or lymph nodes, where infectious prions accumulate in relatively high concentrations. PrP^Sc^ is also present in quantities too low to be detected using classical diagnostic methods in more easily accessible biological samples, so an important limitation is the lack of sensitive *ante-mortem* diagnostic methods.

The application of the RT-QuIC method to Italian wild red deer from the national monitoring plan showed, among all of the samples analyzed, an animal (deer 9) with seeding activity only at the level of the brainstem, differing from the negative results obtained with the official screening methods and confirmatory tests. Although recent diagnostic studies carried out using the validated and authorized tests for the surveillance of cattle and small ruminants have shown them to be valid for detecting European CWD cases [21], the results obtained from this Italian wild deer, on the other hand, given the high specificity of the RT-QuIC method, suggest a significantly higher diagnostic sensitivity compared to traditional TSE diagnostic tests. The failure to detect PrP^Sc^ in this red deer through screening methods is probably due to very low amounts of the etiological agent in the CNS, therefore indicative of a pre-clinical disease condition. The discordant results obtained in this case could also raise several questions about the specificity of this outcome. However, a large number of studies have already demonstrated the very high sensitivity of RT-QuIC, which, unlike classical methods, allows for the detection of the presence of the TSE agent very early in various biological matrices, several months before the onset of clinical symptoms [34,35]. In addition to the elevated sensitivity, many studies have also demonstrated a high specificity of the RT-QuIC method [10,11,12,13]. The sensitivity of RT-QuIC can be influenced by several variables, such as protein extraction methods, preparation of homogenates, and tested biological samples (blood, excreta, and bodily fluids), which can also contain factors inhibiting the in vitro amplification reaction, which is the basic principle of this method [10,11,12,13]. Non-specific results may occur in an RT-QuIC assay due to possible cross-contamination of the samples analyzed with positive material or auto-seeding events of the rPrP substrate, which needs to be prepared and quality-controlled according to precise protocols. To overcome these problems associated with the RT-QuIC assay and to ensure maximum sensitivity and specificity, the analyses were conducted on this Italian wild deer using different batches of rPrP Ha90-231 and BV 23-231, preparing brainstem homogenates from two different samplings and monitoring each analytical session with the inclusion of positive and negative CWD samples. The results of RT-QuIC analyses that showed amplification in CWD-negative controls and/or no amplification in CWD-positive controls were excluded.

The knowledge gained from the scientific literature on the RT-QuIC method presumably points to a real presence of the pathological marker in the nervous system of this wild deer case. The information provided about this wild red deer, although limited, would support this assumption as, on the one hand, this animal belongs to a high-risk category, on which CWD surveillance in Europe is now focused, and on the other hand, it was recovered in an area of Italy where there have been several outbreaks of classical scrapie. A peculiar characteristic of TSEs is their interspecies transmission; it is now well known that the BSE agent can infect a range of animal species (small ruminants, big cats, and mink) as well as humans, overcoming the species barrier. Transmission studies have also shown that intracerebral inoculation of a sheep scrapie agent in wapiti resulted in spongiform encephalopathy with pathological PrP accumulation in the central nervous system [36]. Moreover, the absence of PrP^Sc^ in the medial retropharyngeal lymph node of this Italian deer highlights a pathogenic pattern like that described in Norwegian red deer affected with CWD. However, no connection can be made between the site where this deer was discovered dead and the possible presence of TSEs in this area since, as a wild animal, it is not possible to establish where it came from or where it could have spent most of its life. Additionally, the genetic analysis of the *PRNP* gene from this suspect case supports a genetic background compatible with CWD susceptibility [33].

The observation of seeding activity detected using the RT-QuIC method in a sample does not always correlate with its infectivity and, moreover, considering the importance of this unexpected result in the Italian cervid population, further diagnostic investigations through other highly sensitive methods, such as PMCA, will be needed. However, although this amplification method, unlike RT-QuIC, can provide more detailed information about the possible infectivity and molecular characteristics of the PrP^Sc^ detected in a sample, only a biological assay will confirm the true infectivity of the pathological marker detected in this case.

## 5. Conclusions

This study showed that overall, the results obtained with the HerdChek BSE–Scrapie Antigen Test and RT-QuIC agree except in one case. Our findings highlight the potential of the RT-QuIC method to detect a very low amount of PrP^Sc^-associated seeding activity that may escape detection using classical methods. While seeding activity does not always equate to infectivity, only a bioassay will confirm the real disease status of this Italian case. These findings support the integration of RT-QuIC as a powerful complementary tool within existing surveillance frameworks to strengthen early detection and diagnostic accuracy.

## Figures and Tables

**Table 1:** Results of diagnostic investigations obtained from Italian deer 9.

Sample	RT-QuIC		HerdChek 1st Analysis	HerdChek 2nd Analysis	TeSeE SAP Combi	Confirmatory WB	Confirmatory IHC
	Ha90-231	BV					
brainstem	+	+	N	N	N	N	N
RPLN	-	-	N	N	N	N	N

+: presence of seeding activity; -: absence of seeding activity; N: negative.

**Table 2:** *PRNP* gene analysis of deer 9.

Codon	Allele	Genotype
59	ggc (Gly)	ggc/ggc (G/G)
78	cag (Gln)	cag/cag (Q/Q)
79	ccc (Pro)	ccc/ccc (P/P)
98	acc (Thr)	acc/acc (T/T)
136	gct (Ala)	gct/gct (A/A)
168	cca (Pro)	cca/cca (P/P)
226	cag (Gln)	cag/cag (Q/Q)

## Data Availability

Data are contained within the article and Supplementary Materials.

## Supplementary Materials

The following supporting information can be downloaded at https://www.mdpi.com/article/10.3390/pathogens14101053/s1. Figure S1: RT-QuIC analysis of brainstem homogenates tissue from European CWD cases: Norwegian (N), Finnish (F) and Sweden cases (S); Figure S2: Confirmatory Western Blot analysis of brainstem and medial retropharyngeal lymph node homogenates digested with proteinase K of Italian CWD deer 9, Canadian and Norwegian CWD positive moose; Figure S3: Confirmatory immunohistochemistry analysis on brainstem and retropharyngeal lymph node of CWD positive controls; Figure S4: Confirmatory immunohistochemistry analysis of brainstem and retropharyngeal lymph node of deer 9; Figure S5: RT-QuIC analysis with rPrP^Sen^ 90-231 of brainstem homogenates from the Italian deer 9 treated with and without ethanol.
