# Supplementary figures and images for: Real-Time Quaking-Induced Conversion Assay Applied to the Italian Chronic Wasting Disease Monitoring Plan: Comparison of Classical and Innovative Diagnostic Methods
Source: Pathogens. 2025 Oct 18;14(10):1053. doi: 10.3390/pathogens14101053 (PMC12567053; doi:10.3390/pathogens14101053)

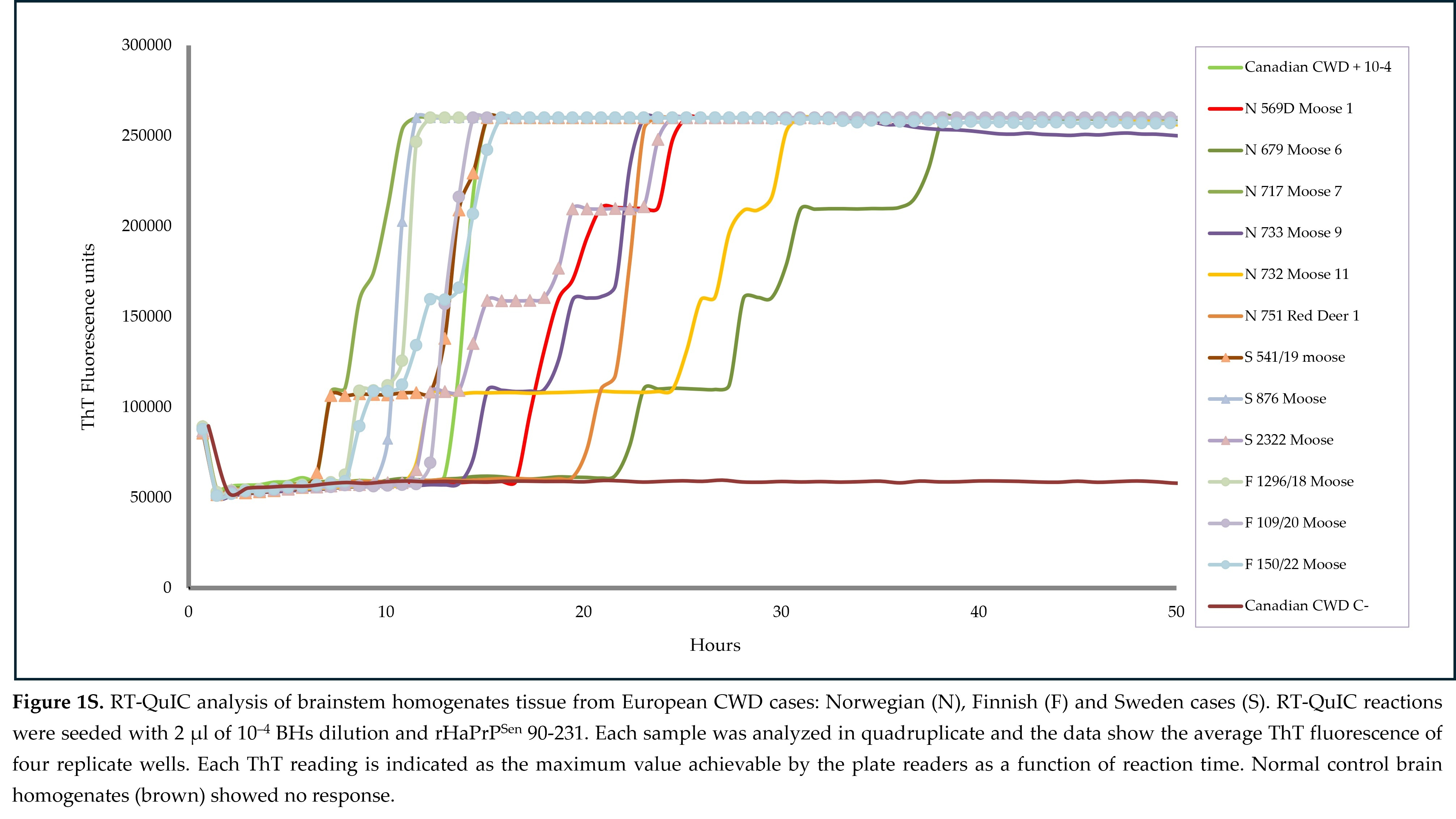

Supplement: Supplementary file 1 [file pathogens-14-01053-s001.zip › Figure 1S.tif]

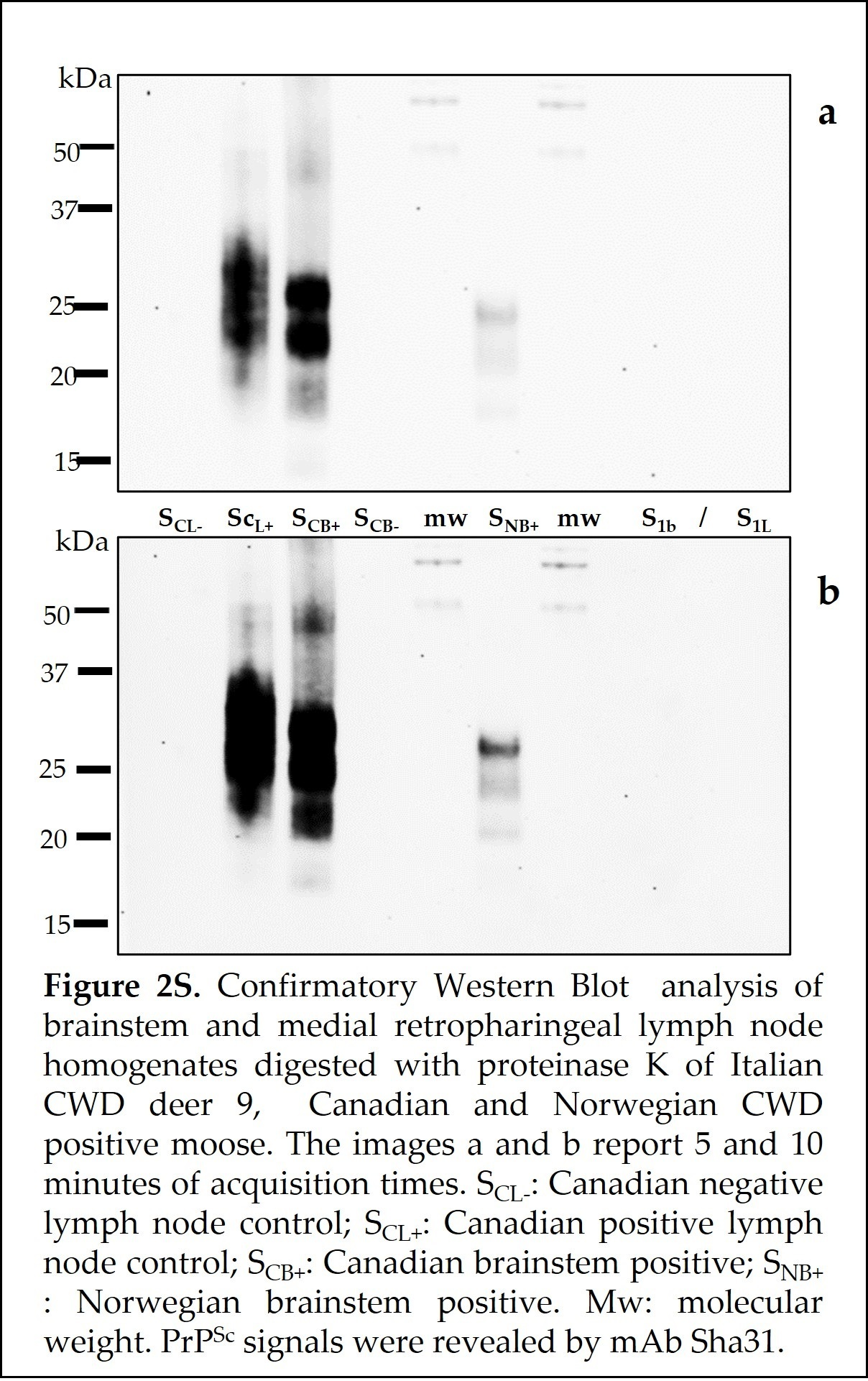

Supplement: Supplementary file 1 [file pathogens-14-01053-s001.zip › Figure 2S.tif]

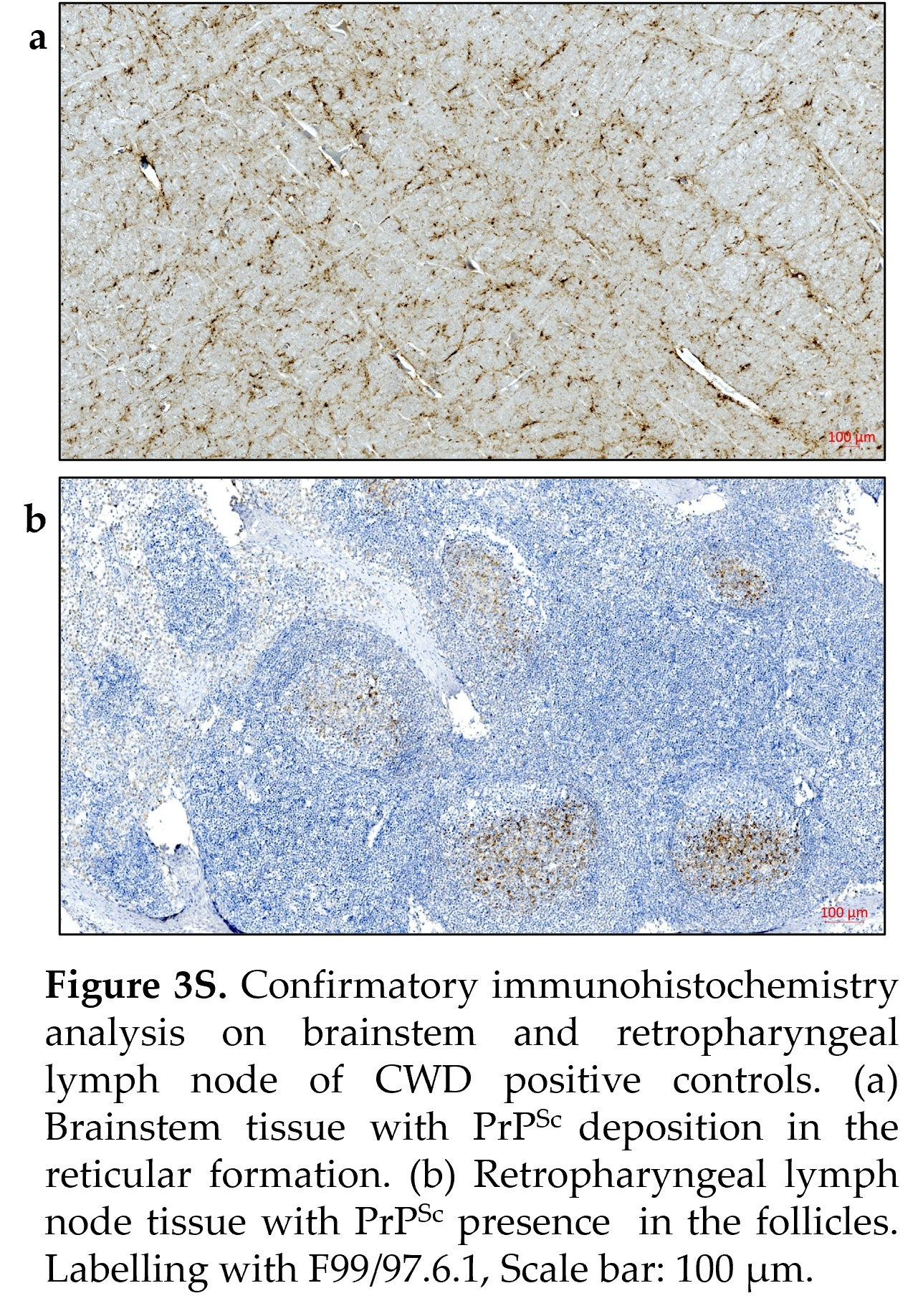

Supplement: Supplementary file 1 [file pathogens-14-01053-s001.zip › Figure 3S.tif]

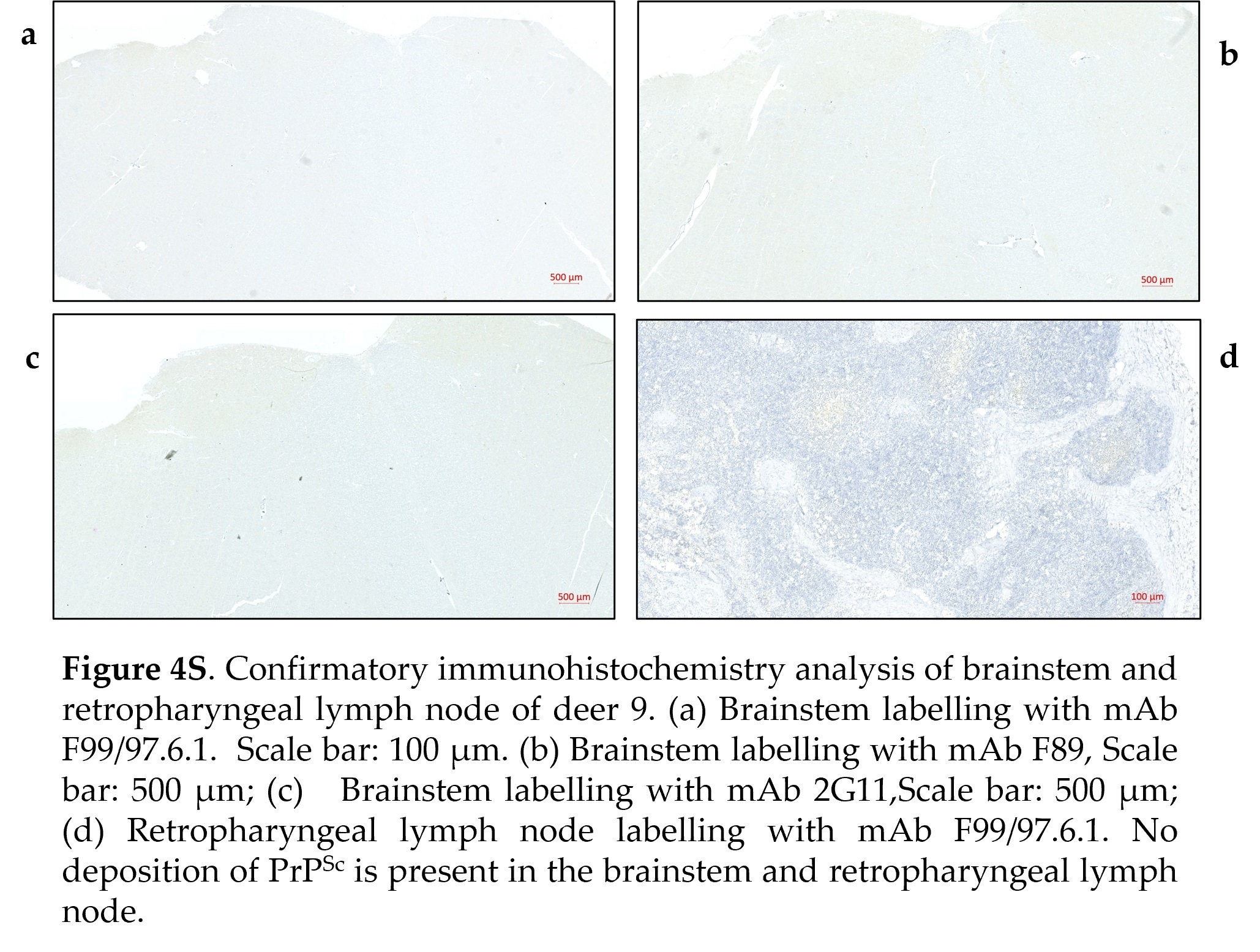

Supplement: Supplementary file 1 [file pathogens-14-01053-s001.zip › Figure 4S.tif]

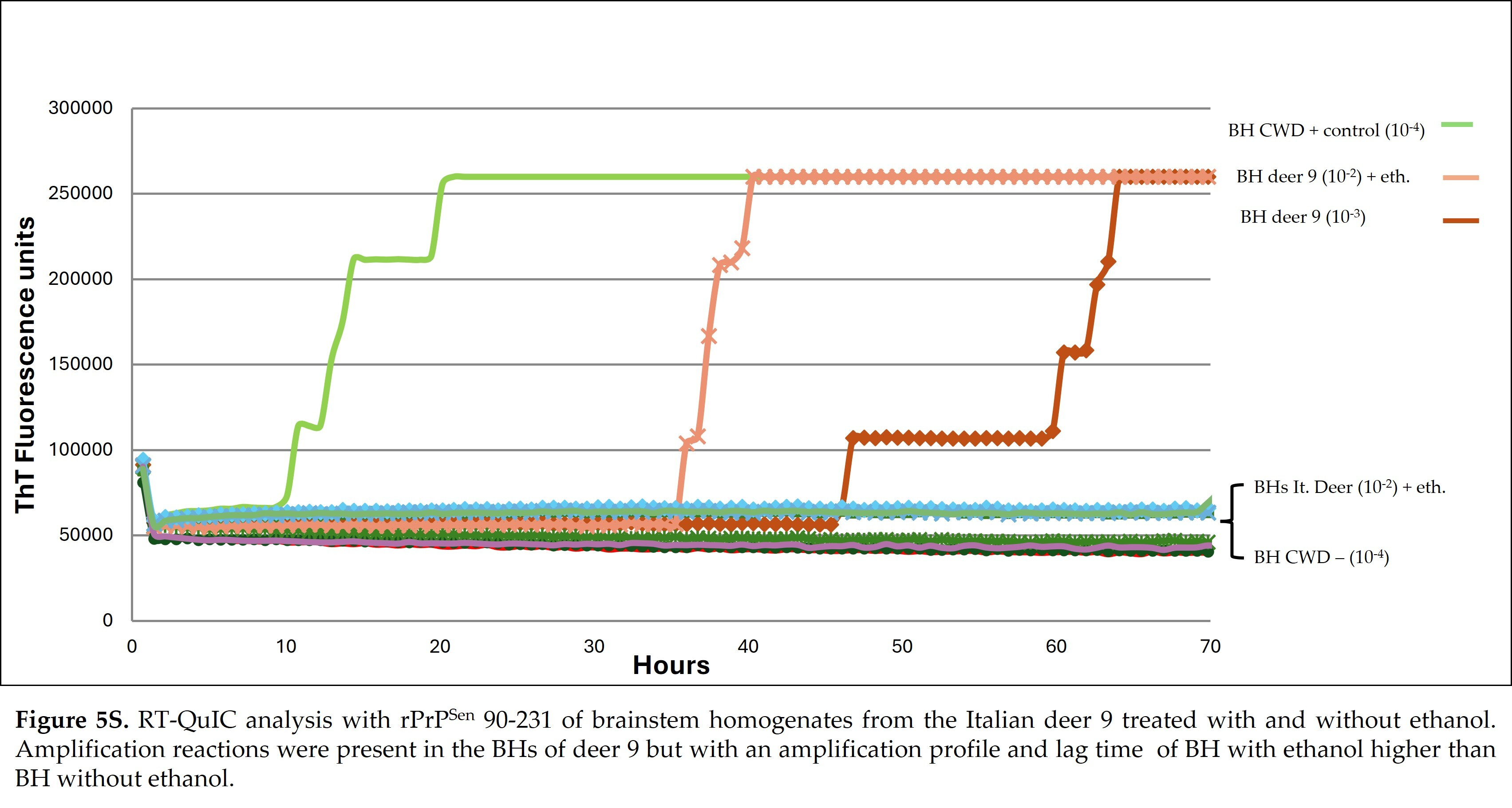

Supplement: Supplementary file 1 [file pathogens-14-01053-s001.zip › Figure 5S.tif]
